# Cardiovascular consequence of reclining vs. sitting beach-chair body position for induction of anesthesia

**DOI:** 10.3389/fphys.2014.00187

**Published:** 2014-05-19

**Authors:** Søren L. Larsen, Tobias S. Lyngeraa, Christian P. Maschmann, Johannes J. Van Lieshout, Frank C. Pott

**Affiliations:** ^1^Department of Anesthesiology, Bispebjerg Hospital, University of CopenhagenDenmark; ^2^Acute Admissions Unit, Laboratory for Clinical Cardiovascular Physiology, Department of Internal Medicine, AMC Center for Heart FailureUniversity of Amsterdam, Netherlands; ^3^Queen's Medical Centre, School of Life Sciences, University of Nottingham Medical SchoolNottingham, UK

**Keywords:** anesthesia, hemodynamics, patient positioning, shoulder, near-infrared spectroscopy

## Abstract

The sitting beach-chair position is regularly used for shoulder surgery and anesthesia may be induced in that position. We tested the hypothesis that the cardiovascular challenge induced by induction of anesthesia is attenuated if the patient is placed in a reclining beach-chair position. Anesthesia was induced with propofol in the sitting beach-chair (*n* = 15) or with the beach-chair tilted backwards to a reclining beach-chair position (*n* = 15). The last group was stepwise tilted to the sitting beach-chair position prior to surgery. Hypotension was treated with ephedrine. Continuous hemodynamic variables were recorded by photoplethysmography and frontal cerebral oxygenation (ScO_2_) by near infrared spectroscopy. Significant differences were only observed immediately after the induction when patients induced in a reclining beach-chair position had higher mean arterial pressure (MAP) (35 ± 12 vs. 45 ± 15 % reduction from baseline, *p* = 0.04) and ScO_2_ (7 ± 6 vs. 1 ± 8% increase from baseline, *p* = 0.02) and received less ephedrine (mean: 4 vs. 13 mg, *p* = 0.048). The higher blood pressure and lower need of vasopressor following induction of anesthesia in the reclining compared to the sitting beach-chair position indicate more stable hemodynamics with the clinical implication that anesthesia should not be induced with the patient in the sitting position.

## Introduction

During endoscopic shoulder surgery the patient is preferably placed in the sitting beach-chair position (Skyhar et al., 1988; Pohl and Cullen, 2005). This position facilitates surgical access (Papadonikolakis et al., 2008; Tange et al., 2010) and limits the blood loss since the shoulder is above heart level (Papadonikolakis et al., 2008).

On the other hand, the sitting position is associated with pooling of blood in the legs. The resulting postural reduction in stroke volume (SV) and cardiac output (CO) impacts the circulation (Dalrymple et al., 1979; Porter et al., 1999; Buhre et al., 2000; Truijen et al., 2012). In healthy awake humans, baroreceptor reflex-mediated sympathetic activation with an increase in heart rate (HR) and vascular tone maintains mean arterial pressure (MAP), but induction of anesthesia with propofol attenuates this adaptive response. The circulatory challenge of being positioned in a sitting position during induction of anesthesia may jeopardize maintenance of MAP leading to bradycardic hypotensive events (Kinsella and Tuckey, 2001; Jeong et al., 2012). Against this background we questioned whether under these circumstances the hemodynamic challenge of anesthesia in the sitting beach-chair position compromises cerebral perfusion and oxygenation (McCulloch et al., 2010; Lee et al., 2011; Moerman et al., 2012).

In a survey among 26 anesthesiology departments in Denmark, ~40% preferred induction of anesthesia for shoulder surgery with the patient positioned in the sitting beach-chair position to reduce the risk of nerve injury by repositioning an anesthetized patient, and to minimize the setup time. Nerve injury is extremely rare in the beach-chair position (Peruto et al., 2009). However, transient or permanent loss of neural conductivity, so-called neurapraxia, may occur due to nerve fiber compression or inadvertent stretch especially when repositioning the head (Rains et al., 2011).

While several studies focused on the perioperative hemodynamic challenge of the beach-chair position (Dalrymple et al., 1979; Porter et al., 1999; Buhre et al., 2000; Jeong et al., 2012; Moerman et al., 2012), no attention has been paid to the position of the patient during induction of anesthesia. It remains unsettled whether induction of anesthesia in the reclining vs. sitting beach-chair position secures cerebrovascular hemodynamics. To that purpose we investigated the effects of induction of anesthesia in the reclining beach-chair position with subsequent stepwise rise to the sitting beach-chair position vs. induction of anesthesia in the sitting beach-chair position on MAP and cerebral oxygenation.

## Materials and methods

### Patients

This quality control study was performed to assess a change in clinical practice after the departments' advisory board had discouraged induction of anesthesia in the sitting beach-chair position, and it was approved by the Ethical Committee of Copenhagen (H-3-2013-FSP15). Data were recorded in 15 consecutive patients in whom anesthesia was induced in the reclining beach-chair position and results compared to those from a historical control group of 15 patients induced in the sitting beach-chair position. These patients had participated in a trial on the effect of a sequential leg compression device on hemodynamic stability during anesthesia in the sitting beach-chair position (ethical approval: H-1-2009-070; registered in Clinical Trials NCT01680393). Apart from the position of the patient during induction of anesthesia, inclusion procedures, the setup, and the investigators were the same for both groups. All patients signed written informed consent prior to the investigation.

Patients undergoing elective shoulder arthroscopy in general anesthesia were eligible for inclusion when >18 years and in ASA physical status I–II. All patients received an interscalene block (ropivacaine 7.5 mg/ml, 10–20 ml) and wore TED compression stockings throughout the surgical procedure. On the day of surgery, patients were allowed to take clear fluids freely until 2 h before the induction of anesthesia. Hemodynamic variables (MAP, HR, SV, and CO), frontal cerebral oxygenation (ScO_2_), and lower leg oxygenation (SmO_2_) were recorded continuously. Primary endpoint was the decrease in MAP, whereas the amount of ephedrine administered, ScO_2_, SmO_2_, HR, SV, and CO were secondary endpoints.

### Study protocol

#### Baseline

For both groups signal recording started with the patients sitting comfortably in the sitting beach-chair. After ~5 min rest, baseline values were registered as 1 min averages.

#### Reclining beach-chair position

After baseline monitoring patients in the reclining beach-chair group were tilted backwards so that the toes were at the same height as the forehead. Following pre-oxygenation anesthesia was induced. When the hemodynamic condition was judged stable (approximately 5–10 min after induction), the patients were tilted ~30° to halfway sitting position prior to sterile draping and remained in that position for approximately 5 min. Prior to the surgical procedure the patients were tilted further up (~60° sitting beach-chair position), and remained in that position throughout the surgical procedure.

#### Sitting beach-chair position

The body position of the sitting group was maintained during baseline signal recording, induction of anesthesia and throughout the surgical procedure.

#### Positioning of beach-chair (sitting)

The surgical table was set into the sitting beach-chair position with the upper body section raised to ~60°, the mid-section in ~10° Trendelenburg position, and the leg section flexed ~20° at the level of the knees. The head was stabilized in a head rest to prevent head rotation which interferes with cerebral blood flow and cerebrovenous drainage (Højlund et al., 2012). The shoulder panel on the operated side was removed, and an arm support was placed on both sides. Straps were fastened around the torso and the legs to fasten the patient.

#### Anesthesia

After the patient was placed, pre-oxygenation started through a loose fitting facial mask and propofol (~0.5 mg kg^−1^ h^−1^) and remifentanil (~0.5 μg kg^−1^ h^−1^) infusions were initiated. Propofol (2.0–2.5 mg kg^−1^ i.v. in a bolus injection) as inductive agent was administered when the patient felt first signs of anesthesia, and a laryngeal mask was placed after loss of eyelid reflexes. Anesthesia was maintained by continued infusion of propofol and remifentanil. Hypotension (MAP<60 mmHg) was treated with ephedrine 5–10 mg. Ventilation was maintained by a respirator with a tidal volume ~8 ml kg^−1^, an inspiratory oxygen fraction of 0.4, and a respiratory frequency ~12 min^−1^. Isotonic saline was administered at ~500 ml h^−1^. Hemodynamic stability was assessed by (1) the maximal decline in MAP, SV and ScO_2_ during the first 4 min following induction of anesthesia and position change; (2) the total dosage of ephedrine administered during anesthesia; and (3) the average change in MAP, HR, SV, and CO under steady state (during surgery, 15 and 30 min following induction of anesthesia) compared with baseline.

### Measurements

#### Oxygenation

ScO_2_ and SmO_2_ were recorded by near-infrared spectroscopy (NIRS, INVOS® System technology, model 5100C, Somanetics Corporation, Troy, MI) (Moritz et al., 2007; Smith and Elwell, 2009). One probe was placed high on the lateral forehead ipsilateral to the arm being operated. A second probe was placed over the left gastrocnemius muscle.

#### Circulatory measurements and data analysis

SV was obtained from continuously measured arterial pressure by the pulse contour method (BMEYE Nexfin® monitor, Amsterdam, The Netherlands) (Martina et al., 2012). A cuff was applied to the midphalanx of the middle finger of the arm not being operated. A “heart reference system” with a transducer at both the finger and the heart level corrected for the hydrostatic difference between the finger cuff and the heart. Compared with Doppler-measured changes in CO, pulse contour analysis provides reliable estimates, especially in regard to changes (Bogert et al., 2010; Van Geldorp et al., 2011; Van der Spoel et al., 2012).

Data from the near-infrared and photoplethysmographic devices were not disclosed to the anesthesiologist who relied on standard intra-operative monitors, including sphygmomanometric blood pressure as measured on the arm opposite to the operated shoulder every second minute following induction and later every fifth minute.

*Post-hoc* analysis included visual judgment of blood pressure tracings for obvious artifacts that were removed using MATLAB 7.12 analysis software (MathWorks, Natick, MA, USA). Signals of ScO_2_, MAP, HR, SV, CO, and SmO_2_ were resampled at 1 Hz and expressed as averages of 15-s intervals.

Blood pressure tracings were inspected for instances of 15-s intervals with hypotension (MAP < 60 mmHg), as were NIRS-tracings for cerebral deoxygenation defined as a 20% decrease in ScO_2_ compared to baseline (Moritz et al., 2007).

### Statistical analysis

Data are expressed as mean ± SD unless otherwise indicated. A sample size of 14 patients in each group gave 80% power to detect a 15% difference in MAP at a significance level of 0.05. Comparison between groups was analyzed using Student's unpaired *t*-test when data were normally distributed; otherwise Mann-Whitney Rank sum test was used. One-Way repeated measurements analysis (ANOVA) was used to test for changes in hemodynamics after shift in body position. Paired Student's *t*-test was used to compare values before and after induction. *P* < 0.05 was considered statistically significant and all statistical procedures were performed using the SigmaPlot version 11.0.

## Results

In two patients (one in each group) photoplethysmographic measurements were of insufficient quality, leaving data from 28 patients for analysis. Patient characteristics were comparable between the two groups (Table 1).

**Table 1:** **Clinical characteristics of the study population**.

	**Sitting beach-chair (*n*** = **14)**	**Reclining beach-chair (*n*** = **14)**
Age (years)	40 ± 17	42 ± 15
Gender (m/f)	9/5	9/5
Height (cm)	175 ± 8	177 ± 8
Weight (kg)	76 ± 11	83 ± 11
BMI (kg/m^2^)	25 ± 3	27 ± 4
Operated side (left/right)	6/8	7/7
Per-operative saline IV (ml)	690 ± 160	660 ± 230
Propofol infusions (mg/kg/h)	0.50 ± 0.11	0.45 ± 0.09
Remifentanil infusions (μg/kg/h)	0.49 ± 0.16	0.44 ± 0.07
Induction bolus of propofol (mg/kg)	2.24 ± 0.39	2.12 ± 0.51

### Prior to induction

Baseline values were similar among the groups (Table 2).

**Table 2:** **Circulatory and oxygenation parameters at baseline, following induction of anesthesia in either the sitting or reclining position, and during subsequent elevation to the sitting position in the patients in whom anesthesia was induced in the reclining position**.

	**Position during induction**	**Baseline**	**Prior to induction (15 s)**	**After induction (minimum 0–4 min)**	**Inclination 30° (minimum 0–4 min)**	**Inclination 60° (minimum 0–4 min)**	**15–30 min following induction**
ScO_2_	Sitting	72 ±, 7 (%)	+7 ±, 5^*^	+1 ±, 8^*^			−4 ±, 10
	Reclining	68 ±, 6 (%)	+12 ±, 4	+7 ±, 6	+3 ±, 15	−5 ±, 12^#^	−1 ±, 10
SmO_2_	Sitting	70 ±, 12 (%)	+2 ±, 7	+11 ±, 6^*^ (Max)			+8 ±, 5
	Reclining	76 ±, 8 (%)	+1 ±, 4	+5 ±, 5 (Max)	+5 ±, 7 (Max)	+8 ±, 6^#^ (Max)	+4 ±, 5
MAP	Sitting	104 ±, 10 (mmHg)	+3 ±, 8	−45 ±, 15^*^			−37 ±, 11
	Reclining	102 ±, 17 (mmHg)	−2 ±, 13	−35 ±, 12	−35 ±, 17	−36 ±, 12	−31 ±, 11
HR	Sitting	73 ±, 16 (beats/min)	+11 ±, 20	−21 ±, 11			−12 ±, 15
	Reclining	71 ±, 16 (beats/min)	+6 ±, 14	−18 ±, 11	−27 ±, 7 ^#^	−24 ±, 9^#^	−21 ±, 10
SV	Sitting	91 ±, 20 (ml)	0 ±, 6	−24 ±, 14			−10 ±, 15
	Reclining	101 ±, 29 (ml)	0 ±, 15	−20 ±, 19	−10 ±, 27	−18 ±, 26	−9 ±, 30
CO	Sitting	6.5 ±, 1.1 (l/min)	+12 ±, 17	−36 ±, 16			−23 ±, 11
	Reclining	6.9 ±, 1.7 (l/min)	+5 ±, 16	−29 ±, 17	−33 ±, 18	−36 ±, 16	−29 ±, 18

All values are mean ± SD. For both groups, absolute baseline values represent a 60 s average in the sitting position. Other values are percent changes compared to baseline; averages of the 15 s interval prior to induction, and the 15–30th min after induction. Between 0 and 4 min after the induction and inclination, values represent the minimum (SmO_2_: maximum) of 15 s averages.

* Sitting vs. reclining; p < 0.05.

# Different from the minimum value after induction; p < 0.05.

In the reclining beach-chair position pre-oxygenation increased ScO_2_ more than in the sitting beach-chair group.

### After induction

**Figure 1:**
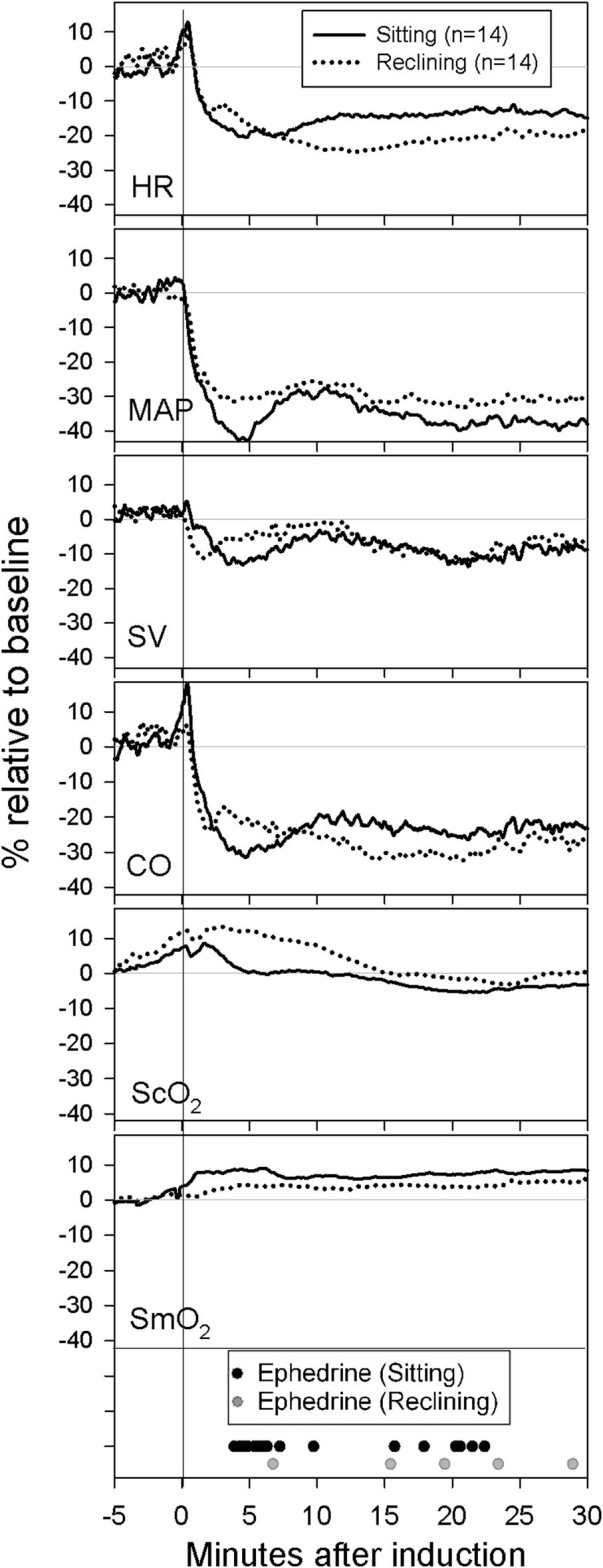
**Mean-values for all patients from 5 min prior to induction until 30 min after induction**. Baseline was recorded in the sitting beach-chair position approximately 5 min prior to induction. During the 5 min prior to induction the patient was placed in the induction position (either sitting or reclining) with start of propofol and remifentanil infusions and preoxygenation. Instances of individual ephedrine-administrations are shown.

Induction of anesthesia resulted in the largest drops in ScO_2_ and MAP in patients induced in the sitting beach-chair position while their SmO_2_ was higher (Table 2). However, due to the pre-oxygenation induced offset, ScO_2_ remained higher than baseline in both groups during the first 4 min after induction of anesthesia. CO tended to be lower (Figure 1), but minimum values for CO, SV, and HR were not different between the groups.

### Tilting to the sitting beach-chair position

When patients from the reclining beach-chair group were tilted to the sitting beach-chair position, MAP, SV, and CO remained stable, whereas HR and ScO_2_ decreased (Table 2). At the same time SmO_2_ increased.

### Changes from baseline to a steady-state condition (15 and 30 min after induction)

In both groups, MAP, HR, SV, and CO were lower 15–30 min after induction of anesthesia without significant differences between the groups (Figure 1).

### Blood pressure and ephedrine treatment

The incidence of hypotensive events was not statistically different between the two groups (Table 3). However, during surgery the group induced in the sitting beach-chair position received more ephedrine (mean: 13 vs. 4 mg, *p* = 0.048), especially during the first 10 min after induction [10 (0–20 mg); median (range) vs. 0 (0–5 mg); *p* < 0.001].

**Table 3:** **Number of 15 s periods per patient with MAP<60 mmHg, (median and range); and the number of ephedrine (Eph) administrations during three consecutive 10 min intervals**.

	**0–10 min**	**10–20 min**	**20–30 min**
	**after induction**	**after induction**	**after induction**
Sitting	13 (0–29); Eph × 11	3 (0–37); Eph × 2	8 (0–34); Eph × 4
Reclining	0 (0–31); Eph × 1	0 (0–40); Eph × 2	2 (0–40); Eph × 2

### Cerebral deoxygenation

Two patients induced in the sitting beach-chair position and three patients in the reclining beach-chair position had episodes with cerebral deoxygenation, defined as a 20% decrease compared to baseline. Cerebral deoxygenations were detected following 10–30 min after induction. In these patients deoxygenations coincided with hypotensive events where MAP decreased by 40–70%.

## Discussion

The main new finding is that induction of anesthesia in the reclining beach-chair position resulted in higher MAP, fewer requirements for ephedrine, and higher ScO_2_ as compared to induction in the sitting beach-chair position.

The observed differences between the two modes of induction are small but may become clinically significant for patients with less effective cerebrovascular autoregulatory capacity associated with microvascular disease in whom any reduction in MAP is translated into a fall in cerebral blood flow (Kim et al., 2011). Following induction of anesthesia ScO_2_ was slightly higher in the reclining compared with the sitting beach-chair group whereas the opposite occurred for SmO_2_. This might be due to caudal accumulation of blood in the sitting position and suggesting that anesthesia induction in the reclining vs. the sitting beach-chair position secures central hemodynamics more efficiently. NIRS recordings might have been different with use of vasopressors other than ephedrine. With phenylephrine and norepinephrine reductions in NIRS signals have been observed concomitant with elevated MAP and were taken to reflect either cerebral vasoconstriction or reduced cardiac stroke volume (Brassard et al., 2009; Nissen et al., 2009). However, recent studies suggest that such reduction is explained by a major contribution of (reduced) skin perfusion to the NIRS signal rather than actual changes in cerebral blood flow (Sørensen et al., 2012, 2014; Ogoh et al., 2014).

No study has addressed the cardio- and cerebrovascular effects of the postural reduction in central blood volume (Buhre et al., 2000; Tange et al., 2010) associated with the sitting position for induction of anesthesia. We can only speculate whether patients induced in the sitting position are imposed to a higher risk of adverse neurologic events, but no such events were reported in >5000 patients who were anesthetized in the supine position and subsequently tilted to the sitting beach-chair position (Pin-On et al., 2013).

In our small number of patients no incidents of post-operative neural dysfunction were observed and the risk of neurapraxia is not expected to be higher by reclining the operation table since the conscious patients place themselves comfortable in the beach-chair including the head-rest. Thus, no major repositioning is performed in anesthetized patients. Although not assessed in this study setup time is expected to be slightly higher (~5 min) when inducing the patient in the reclining position since the subsequent tilting to the sitting position is performed slowly.

We adopted a threshold for cerebral ischemia of 20% change from baseline and during surgery in only five patients desaturations were observed. This may reflect that following induction of anesthesia arterial blood pressure was not reduced below the lower limit of the cerebral autoregulation (Joshi et al., 2012). However, the evolving concept of the brain as a index organ is ambiguous, so it is relevant preventing hypotension because other organs, e.g., the kidneys, may suffer from comprised perfusion before the brain becomes affected due to the hierarchy of blood flow (Ono et al., 2013). As even brief hypotensive episodes may predispose patients to postoperative complications (Fischer et al., 2011) prompt reversal of ScO_2_ in those patients is crucial to improve clinical outcome (Casati et al., 2005).

During induction in the sitting vs. reclining beach-chair group the hemodynamic challenge is larger by the caudal accumulation of blood and in turn reduced cardiac preload. However, in our small group of patients changes in SV and CO were not consistent albeit there was a tendency for lower values following induction of anesthesia with the upper body elevated. Of interest, HR tended to be higher, which may equally reflect the more frequent use of ephedrine as well as strain on the circulation.

Following induction of anesthesia ScO_2_ was higher in the reclining compared with the sitting beach-chair group whereas the opposite occurred for SmO_2_ indicating caudal accumulation of blood in the sitting position. Such gravitational influence is supported by similar changes in SmO_2_ when patients induced in the reclining beach-chair subsequently were tilted to the sitting position. These results are in line with those observed in conscious volunteers during head up tilt, where a rapid increase in the concentration of oxygenated hemoglobin (HbO_2_) of the calf reflects an initially rapid arterial inflow into the leg (Truijen et al., 2012). A subsequent postural reduction in HbO_2_ may represent reflex vasoconstriction, as a decrease in HbO_2_ correlates with leg blood flow and inversely with sympathetic activity (Hachiya et al., 2010). However, following induction of anesthesia more pronounced and opposite changes in both muscle oxygenation and blood pressure in the sitting group suggest that anesthesia attenuates counter-regulatory mechanisms to orthostasis. Of interest, during beach-chair surgery intermittent pneumatic sequential compression of the lower extremities stabilizes hemodynamics (Kwak et al., 2010).

### Limitations

The reclining beach-chair group received less ephedrine although the incidence of significant hypotension was similar in the two groups. Apparently, the anesthetists might have had a lower threshold for the use of ephedrine in the sitting beach-chair group. However, even with less ephedrine treatment, the patients induced in the reclining beach-chair position had higher MAP than the patients induced in the sitting beach-chair position.

Following an interscalene block, local anesthetics may spread to the stellate ganglion (Song and Roh, 2012), and especially a right stellate ganglion block may suppress cardiac sympathetic function (Koyama et al., 2002). Since the side of blockade was evenly distributed within each group we consider this effect unlikely to explain the hemodynamic differences.

## Conclusions

Induction of anesthesia in the reclining compared with the sitting beach-chair position resulted in higher MAP and ScO_2_ as well as less frequent use of ephedrine indicating more stable hemodynamics. We propose that for surgery in the beach-chair position, induction of anesthesia is performed in the reclining position with the chair tilted backward.

## Funding

This work was supported by the Lundbeck-foundation Denmark, (grant number R83-A8124).

### Conflict of interest statement

The authors declare that the research was conducted in the absence of any commercial or financial relationships that could be construed as a potential conflict of interest.

## References

[B1] Bogert L. W. J. Wesseling K. H. Schraa O. Van Lieshout E. J. De Mol B. A. J. M. Van Goudoever J. (2010). Pulse contour cardiac output derived from non−invasive arterial pressure in cardiovascular disease. Anaesthesia 65, 1119–1125 10.1111/j.1365-2044.2010.06511.x 20860647

[B2] Brassard P. Seifert T. Secher N. H. (2009). Is cerebral oxygenation negatively affected by infusion of norepinephrine in healthy subjects? Br. J. Anaesth. 102, 800–805 10.1093/bja/aep065 19376788

[B3] Buhre W. Weyland A. Buhre K. Kazmaier S. Mursch K. Schmidt M. (2000). Effects of the sitting position on the distribution of blood volume in patients undergoing neurosurgical procedures. Br. J. Anaesth. 84, 354–357 10.1093/oxfordjournals.bja.a013439 10793596

[B4] Casati A. Fanelli G. Pietropaoli P. Proietti R. Tufano R. Danelli G. (2005). Continuous monitoring of cerebral oxygen saturation in elderly patients undergoing major abdominal surgery minimizes brain exposure to potential hypoxia. Anesth. Analg. 101, 740–747 10.1213/01.ane.0000166974.96219.cd 16115985

[B5] Dalrymple D. G. Macgowan S. W. Macleod G. F. (1979). Cardiorespiratory effects of the sitting position in neurosurgery. Br. J. Anaesth. 51, 1079–1082 10.1093/bja/51.11.1079 518805

[B6] Fischer G. W. Lin H.-M. Krol M. Galati M. F. Di Luozzo G. Griepp R. B. (2011). Noninvasive cerebral oxygenation may predict outcome in patients undergoing aortic arch surgery. J. Thorac. Cardiovasc. Surg. 141, 815–821 10.1016/j.jtcvs.2010.05.017 20579669

[B7] Hachiya T. Walsh M. L. Saito M. Blaber A. P. (2010). Delayed vasoconstrictor response to venous pooling in the calf is associated with high orthostatic tolerance to LBNP. J. Appl. Physiol. 109, 996–1001 10.1152/japplphysiol.00593.2009 20651224

[B8] Højlund J. Sandmand M. Sonne M. Mantoni T. Jørgensen H. L. Belhage B. (2012). Effect of head rotation on cerebral blood velocity in the prone position. Anesthesiol. Res. Pract. 2012, 647258 10.1155/2012/647258 22988456 PMC3440850

[B9] Jeong H. Jeong S. Lim H. J. Lee J. Yoo K. Y. (2012). Cerebral oxygen saturation measured by near-infrared spectroscopy and jugular venous bulb oxygen saturation during arthroscopic shoulder surgery in beach chair position under sevoflurane-nitrous oxide or propofol-remifentanil anesthesia. Anesthesiology 116, 1047–1056 10.1097/ALN.0b013e31825154d2 22421420

[B10] Joshi B. Ono M. Brown C. Brady K. Easley R. B. Yenokyan G. (2012). Predicting the limits of cerebral autoregulation during cardiopulmonary bypass. Anesth. Analg. 114, 503–510 10.1213/ANE.0b013e31823d292a 22104067 PMC3288415

[B11] Kim Y.-S. Davis S. C. A. T. Truijen J. Stok W. J. Secher N. H. van Lieshout J. J. (2011). Intensive blood pressure control affects cerebral blood flow in type 2 diabetes mellitus patients. Hypertension 57, 738–745 10.1161/HYPERTENSIONAHA.110.160523 21357278

[B12] Kinsella S. M. Tuckey J. P. (2001). Perioperative bradycardia and asystole: relationship to vasovagal syncope and the Bezold–Jarisch reflex. Br. J. Anaesth. 86, 859–868 10.1093/bja/86.6.859 11573596

[B13] Koyama S. Sato N. Nagashima K. Aizawa H. Kawamura Y. Hasebe N. (2002). Effects of right stellate ganglion block on the autonomic nervous function of the heart: a study using the head-up tilt test. Circ. J. 66, 645–648 10.1253/circj.66.645 12135131

[B14] Kwak H. J. Lee J. S. Lee D. C. Kim H. S. Kim J. Y. (2010). The effect of a sequential compression device on hemodynamics in arthroscopic shoulder surgery using beach-chair position. Arthrosc. J. Arthrosc. Relat. Surg. 26, 729–733 10.1016/j.arthro.2009.10.001 20511029

[B15] Lee J. H. Min K. T. Chun Y.-M. Kim E. J. Choi S. H. (2011). Effects of beach-chair position and induced hypotension on cerebral oxygen saturation in patients undergoing arthroscopic shoulder surgery. Arthroscopy 27, 889–894 10.1016/j.arthro.2011.02.027 21620637

[B16] Martina J. R. Westerhof B. E. van Goudoever J. de Beaumont E. M. F. H. Truijen J. Kim Y.-S. (2012). Noninvasive continuous arterial blood pressure monitoring with Nexfin®. Anesthesiology 116, 1092–1103 10.1097/ALN.0b013e31824f94ed 22415387

[B17] McCulloch T. J. Liyanagama K. Petchell J. (2010). Relative hypotension in the beach-chair position: effects on middle cerebral artery blood velocity. Anaesth. Intensive Care 38, 486–491 20514957 10.1177/0310057X1003800312

[B18] Moerman A. T. De Hert S. G. Jacobs T. F. De Wilde L. F. Wouters P. F. (2012). Cerebral oxygen desaturation during beach chair position. Eur. J. Anaesthesiol. 29, 82–87 10.1097/EJA.0b013e328348ca18 21730865

[B19] Moritz S. Kasprzak P. Arlt M. Taeger K. Metz C. (2007). Accuracy of cerebral monitoring in detecting cerebral ischemia during carotid endarterectomy. Anesthesiology 107, 563–569 10.1097/01.anes.0000281894.69422.ff 17893451

[B20] Nissen P. Brassard P. Jørgensen T. B. Secher N. H. (2009). Phenylephrine but not ephedrine reduces frontal lobe oxygenation following anesthesia-induced hypotension. Neurocrit. Care 12, 17–23 10.1007/s12028-009-9313-x 19957053

[B21] Ogoh S. Sato K. Okazaki K. Miyamoto T. Secher F. Sorensen H. (2014). A decrease in spatially resolved near-infrared spectroscopy-determined frontal lobe tissue oxygenation by phenylephrine reflects reduced skin blood flow. Anesth. Analg. 118, 823–829 10.1213/ANE.0000000000000145 24651237

[B22] Ono M. Arnaoutakis G. J. Fine D. M. Brady K. Easley R. B. Zheng Y. (2013). Blood pressure excursions below the cerebral autoregulation threshold during cardiac surgery are associated with acute kidney injury. Crit. Care Med. 41, 464–471 10.1097/CCM.0b013e31826ab3a1 23263580 PMC3769417

[B23] Papadonikolakis A. Wiesler E. R. Olympio M. A. Poehling G. G. (2008). Avoiding catastrophic complications of stroke and death related to shoulder surgery in the sitting position. Arthroscopy 24, 481–482 10.1016/j.arthro.2008.02.005 18375282

[B24] Peruto C. M. Ciccotti M. G. Cohen S. B. (2009). Shoulder arthroscopy positioning: lateral decubitus versus beach chair. Arthroscopy 25, 891–896 10.1016/j.arthro.2008.10.003 19664509

[B25] Pin-On P. Schroeder D. Munis J. (2013). The hemodynamic management of 5177 neurosurgical and orthopedic patients who underwent surgery in the sitting or “beach chair” position without incidence of adverse neurologic events. Anesth. Analg. 116, 1317–1324 10.1213/ANE.0b013e31828446bb 23477958

[B26] Pohl A. Cullen D. J. (2005). Cerebral ischemia during shoulder surgery in the upright position: a case series. J. Clin. Anesth. 17, 463–469 10.1016/j.jclinane.2004.09.012 16171668

[B27] Porter J. M. Pidgeon C. Cunningham A. J. (1999). The sitting position in neurosurgery: a critical appraisal. Br. J. Anaesth. 82, 117–128 10.1093/bja/82.1.117 10325848

[B28] Rains D. D. Rooke G. A. Wahl C. J. (2011). Pathomechanisms and complications related to patient positioning and anesthesia during shoulder arthroscopy. Arthroscopy 27, 532–541 10.1016/j.arthro.2010.09.008 21186092

[B29] Skyhar M. J. Altchek D. W. Warren R. F. Wickiewicz T. L. O'Brien S. J. (1988). Shoulder arthroscopy with the patient in the beach-chair position. Arthroscopy 4, 256–259 3233114 10.1016/s0749-8063(88)80040-9

[B30] Smith M. Elwell C. (2009). Near-infrared spectroscopy: Shedding light on the injured brain. Anesth. Analg. 108, 1055–1057 10.1213/ane.0b013e31819a0301 19299760

[B31] Song S. Y. Roh W. S. (2012). Hypotensive bradycardic events during shoulder arthroscopic surgery under interscalene brachial plexus blocks. Korean J. Anesthesiol. 62, 209–219 10.4097/kjae.2012.62.3.209 22474545 PMC3315648

[B32] Sørensen H. M. S. Secher N. H. M. D. Siebenmann C. M. S. Nielsen H. B. M. D. Kohl-Bareis M. P. D. Lundby C. P. D. (2012). Cutaneous vasoconstriction affects near-infrared spectroscopy determined cerebral oxygen saturation during administration of norepinephrine. Anesthesiology 117, 263–270 10.1097/ALN.0b013e3182605afe 22739762

[B33] Sørensen H. Rasmussen P. Sato K. Persson S. Olesen N. D. Nielsen H. B. (2014). External carotid artery flow maintains near infrared spectroscopy-determined frontal lobe oxygenation during ephedrine administration. Br. J. Anaesth. [Epub ahead of print]. 10.1093/bja/aet481 24508985

[B34] Tange K. Kinoshita H. Minonishi T. Hatakeyama N. Matsuda N. Yamazaki M. (2010). Cerebral oxygenation in the beach chair position before and during general anesthesia. Minerva Anestesiol. 76, 485–490 20613688

[B35] Truijen J. Kim Y. S. Krediet C. T. P. Stok W. J. Kölgen R. S. Colier W. N. (2012). Orthostatic leg blood volume changes assessed by near-infrared spectroscopy. Exp. Physiol. 97, 353–361 10.1113/expphysiol.2011.061051 22090063

[B36] Van der Spoel A. G. E. Voogel A. J. Folkers A. Boer C. Bouwman R. A. (2012). Comparison of noninvasive continuous arterial waveform analysis (Nexfin) with transthoracic Doppler echocardiography for monitoring of cardiac output. J. Clin. Anesth. 24, 304–309 10.1016/j.jclinane.2011.09.008 22608585

[B37] Van Geldorp I. E. Delhaas T. Hermans B. Vernooy K. Broers B. Klimusina J. (2011). Comparison of a non-invasive arterial pulse contour technique and echo Doppler aorta velocity-time integral on stroke volume changes in optimization of cardiac resynchronization therapy. Europace 13, 87–95 10.1093/europace/euq348 20880954

